# Down-regulation of MIR-378A-3P expression associated with inflammation: The effects of restoring its levels

**DOI:** 10.1371/journal.pone.0329685

**Published:** 2025-08-11

**Authors:** Marta Seco-Cervera, Laura Gisbert-Ferrándiz, Dulce C. Macias-Ceja, Dolores Ortiz-Masiá, Jesús Cosín-Roger, Cristina Bauset, Begoña Heras-Moran, Francisco Navarro-Vicente, Maria Civera-Barrachina, José Santiago Ibáñez-Cabellos, Sara Calatayud, María D. Barrachina

**Affiliations:** 1 Hospital Universitario Dr. Peset, FISABIO, Valencia, Spain; 2 Departamento de Farmacología, Facultad de Medicina, Universidad de Valencia, Valencia, Spain; 3 Departamento de Medicina, Facultad de Medicina, Universidad de Valencia, Valencia, Spain; 4 CIBERehd, Valencia, Spain; 5 Departamento de Anatomía Patológica, Facultad de Medicina, Universidad de Valencia, Valencia, Spain; 6 Hospital de Manises, Valencia, Spain; 7 EpiDisease S.L., Scientific Park, University of Valencia, Departamento de Fisiología, Facultad de Medicina, Universidad de Valencia, Valencia, Spain; University of Pennsylvania, UNITED STATES OF AMERICA

## Abstract

Epigenetics has emerged as a modulator of inflammation-related diseases and changes in miRNA expression have been associated with regional location, inflamed mucosa and disease activity in Crohn´s disease (CD). We analyse here the differential ileal miRNA expression in fibrotic tissue from patients with complicated CD and its relevance in inflammation and fibrosis. A miRNA sequencing analysis has been performed in ileal surgical resections from both patients with complicated CD and control subjects. The correlation analysis of data with an mRNA seq study performed in the same samples pointed to hsa-miR-378a-3p as an epigenetic regulator of inflammatory and fibrotic genes. Results demonstrate a significant diminution in the expression of miR-378a-3p in three different inflammatory conditions: ileum from complicated CD patients, intestine from DSS (Dextran Sulfate Sodium)-treated mice and macrophages polarized towards an M1 phenotype. Treatment with miR-378a-3p mimics failed to prevent inflammation and fibrosis in DSS-treated mice while it increased the expression of several cytokines and chemokines in both murine intestine and M1 macrophages. In conclusion, our study shows the downregulation of miR-378a-3p expression in human and murine intestinal inflammation and demonstrates that restoring the intestinal miR-378a-3p levels did not prevent inflammation and fibrosis in murine chronic colitis while intensified the expression of inflammatory and fibrotic markers.

## Introduction

Crohn´s disease (CD) is a chronic inflammatory disorder of the gastrointestinal tract that commonly affect the terminal ileum and proximal colon. Ileal CD constitute a risk factor to develop complications towards stenotic or penetrating behaviours [1–3]. An excessive deposition of the extracellular matrix (ECM), has been detected in both complications [4,5], and this fibrotic process is intimately related to chronic inflammation, but inflammation-independent mechanisms involved in the perpetuation of fibrosis have also been reported [6]. Currently, no treatment is available to prevent nor cure intestinal fibrosis and the management of CD complications constitutes a critical challenge.

Epigenetics or the control of gene expression in response to environmental factors has emerged as a modulator of inflammation-related diseases [7,8]. miRNA are small, single-stranded, non-coding RNA molecules which cause post-transcriptional gene silencing or mRNA degradation, and they are among the epigenetics mechanisms regulating CD etiopathogenesis [9,10]. Changes in miRNA expression have been detected in tissue biopsies, circulating body fluids, or stools of patients with CD and they have been associated to several characteristics of the disease: the regional location [11], the inflamed mucosa [12,13], the disease activity [14], the post-surgical recurrence [15] or the stenotic tissue [16]. Each of these conditions is associated with a specific pattern of miRNA expression but, only few miRNA such as, miR-16, miR-155, and miR21s have been consistently altered by all of them [9] which strongly reinforces the relevance of the local microenvironment in epigenetic regulation beyond the pathology itself.

Transmural inflammation is an important feature of CD, but little is known about the pattern of miRNA expression in deep layers of the intestinal wall which are affected by this condition [16–19]. In the present study, to better understand the epigenetic mechanisms that may be modulating gene expression in patients with complicated CD, we performed a miRNA sequencing study in surgical resections including the whole thickness of the ileal wall. Our results demonstrate the diminution of miR-378a-3p levels in the ileum of complicated CD, in the intestine of chronic DSS-treated mice and in macrophages polarized towards an inflammatory phenotype. Restoring the decreased levels of miR-378a-3p in the last two cases did not prevent intestinal inflammation.

## Materials and methods

### Human biological samples

In the present study we perform a miRNA seq analysis in human ileal samples from both patients with complicated CD and non-IBD (inflammatory bowel diseases) subjects (S1 Table) which were used in a previous RNA sequencing study [20]. In the results section, we will include an unpublished comparative analysis of the mRNA expression profile in the same samples. Recruitment of surgical resections happened between May 5, 2021, and April 30, 2023. The study was conducted in accordance with the Declaration of Helsinki and approved by the Institutional Review Board (or Ethics Committee) of Hospital of Manises (Nº de registro: 2021-284-1) located in Valencia, Spain. The privacy rights of human subjects were observed and written informed consent was obtained from all patients.

### Small-RNA sequencing

Approximately 35 mg of frozen intestinal resection samples were utilized for total miRNA extraction, following the manufacturer’s protocol for the miRvana miRNA Isolation Kit (Applied Biosystems/Ambion, USA) as we previously reported [20]. Library preparation and next-generation sequencing of small RNAs were carried out at the Genomics and Epigenetics Section of the Central Unit for Research in Medicine (UCIM) at the University of Valencia, utilizing Illumina sequencing technology.

For small RNA sequencing from ileal samples, libraries were generated with the NEXTFLEX® Small RNA-Seq v3 Kit for Illumina Platforms (Bioo Scientific® Corporation, TX, USA), following the manufacturer’s instructions. Briefly, small RNA molecules were ligated to adenylated 3’-4N adapters targeting the terminal phosphate group to specifically select these molecules. Then, 5’-4N adapters were ligated, followed by reverse transcription to synthesize cDNA. These cDNA fragments were then PCR-amplified and indexed with barcoded primers unique to each sample. The final library preparation step involved size selection. The quality and concentration of the libraries were verified using high-sensitivity DNA chips on the Agilent 2100 Bioanalyzer (Agilent Technologies, USA). An equimolar pool of all samples was then sequenced on the NextSeq550 platform (Illumina, CA, USA) using 50 bp single-end sequencing.

### Small RNA sequencing analysis

The bioinformatics analyses were carried out by Epidisease S.L. mRNA bioinformatic analysis was performed as described previously [20]. Small RNA sequencing data yielded fastq files, which were initially quality-checked with FastQC v0.11.9 [21]. Adapter trimming was conducted on these fastq files using Cutadapt v1.18 [22] and 3’ adapters were removed from miRNA reads. Only miRNA reads with a minimum mean quality score of 20 and a length of at least 18 nucleotides were retained. MultiQC v1.11 [23] was used to summarize sequence quality metrics, including mean Phred scores above 34 and adapter content below 1% for trimmed sequences. Reads were aligned to the human reference genome (ChGR38) using Subread v2.0.2 [24], with mapping rates averaging 84.2% for miRNA reads. miRNAs were annotated using miRBase Database (v22) [25], and read quantification was completed with the Rsubread R-package [26].

Differential expression analysis (DEA) between sample groups was performed on miRNA data. Since small RNA sequencing generally yields low counts, miRNA DEA was conducted with the edgeR R-package. Before the analysis, we excluded miRNAs with no detectable expression to minimize noise [27]. Raw library sizes were scaled using TMM normalization in edgeR for miRNAs. Candidate miRNAs were classified by fold change direction for each comparison, and those with an FDR < 0.05 [28] were considered significant. Hierarchical clustering heatmaps of significant miRNAs were generated in R with the gplots and stats packages, using the heatmap.2 and hclust functions, respectively. Data were normalized to a −1 – 1 scale to enhance visualization.

### Cell culture

U937 human monocytes (European Collection of Cell Culture, Salisbury, UK) were differentiated into macrophages and polarized towards an M1 phenotype as previously described [29]. M1-polarized macrophages were transfected for 24 hours using Lipofectamine™ RNAiMAX with 50 nM mirVana® miRNA mimic for hsa-miR-378a-3p (Cat: 4464066 ID: MC11360) or mirVana™ miRNA Mimic, Negative Control #1 (4464058).

Human Small Intestinal fibroblasts (HSIF) (Innoprot, Derio, Spain) between passages 6 and 10 were cultured and treated as previously reported [20]. Additionally, cells were transfected for 24 hours using Lipofectamine™ RNAiMAX and 20 nM mirVana® miRNA mimic for hsa-miR-378a-3p (Cat: 4464066 ID: MC11360) or mirVana™ miRNA Mimic, Negative Control #1 (4464058).

### Murine chronic colitis induced by DSS

C57Bl/6 female mice, 6–8-week-old received vehicle or Dextran Sulfate Sodium (DSS, 40 kDa, Sigma-Aldrich, St. Louis, MO, USA) for 2 cycles (7 days drinking DSS 2% in water solution followed by 10 days drinking water). DSS is a negatively charged sulfated polysaccharide with a high molecular weight that has the ability to damage the epithelial monolayer lining the large intestine which results in the activation of the immune system, in a similar manner to human colitis [30]. Some mice were intravenously injected with Invivofectamine™ 3.0 Reagent (IVF3001) and 2,5 mg/kg of mirVana™ miRNA Mimic, Negative Control (4464061) (NC-mimics) or mirVana® miRNA mimic for mmu-miR-378a-3p (Cat: 4464070; ID: MC12581) (mmu-miR-378a-3p mimics), twice a week. Body weight and DAI score was obtained every day and according with the protocol, in the event that an animal shows symptoms of extreme suffering, the animal is sacrificed by cervical dislocation. On day 34, mice were properly handled and euthanized by cervical dislocation, and colon tissue samples were collected for further analysis. The animal study protocol was approved by the Institutional Review Board (or Ethics Committee) of University of Valencia performed in compliance with the European Animal Research Law (European Communities Council Directives 2010/63/EU, 90/219/EEC, Regulation (EC) No. 1946/2003), and Generalitat Valenciana (Artículo 31, Real Decreto 53/2013) (Code number: 2021/VSC/PEA/0244).

### Histological analysis

Histological analysis was performed in murine colon samples fixed and embedded in paraffin, sectioned (5µm) and stained with hematoxylin or syrius red as previously described [31]. Histological damage was analyzed in hematoxylin-stained slides by the parameters of Obermeier et al. [32]. Sirius Red staining was performed to determine the collagen deposition, represented by red coloration which was quantified by measuring the thickness of the collagen layer with ImageJ (National Institutes of Health, Bethesda, MD, USA). The measurements were performed in a blinded manner.

### RT-qPCR analysis

To quantify the relative expression of each gene, 1 µg of total RNA was reverse transcribed using the PrimeScript RT Reagent Kit (Takara Biotechnology). Real-time PCR was conducted with the PrimeScript Reagent Kit Perfect Real Time (Takara Biotechnology) on a LightCycler thermocycler (Roche Diagnostics). Specific primers were designed based on sequences provided in S2 Table. To verify that the fragment sizes were correct, PCR products were loaded onto an agarose gel and bands were visualized using a LAS-3000 imaging system (Fujifilm). The Actin Beta (*ACTB* or *Actb*) gene was used as a normalization control for mRNA expression data.

For miRNA relative expression quantification, reverse transcription reactions were performed with the TaqMan miRNA Reverse Transcription Kit and miRNA-specific stem-loop primers (Part No. 4366597, Applied Biosystems, CA, USA) using 100 ng of total RNA in a 20 µL reaction. Real-time PCR reactions were carried out in 10 µL volumes with 5 µL TaqMan 2x Universal PCR Master Mix (Applied Biosystems, CA, USA) without UNG, 0.5 µL of TaqMan Small RNA assay (20x) (Applied Biosystems, CA, USA) [hsa-miR-378a-3p (001314) and mmu-miR-378a-3p (002243)], 3.5 µL of nuclease-free water, and 1 µL of RT product. PCR was performed on a QuantStudio™ 5 Real-Time PCR System (Applied Biosystems, CA, USA) under the following conditions: 50°C for 2 minutes, 95°C for 10 minutes, followed by 45 cycles of 95°C for 15 seconds and 60°C for 1 minute. For normalization, RNU48 (001006) was used for human samples, and snoRNA234 (001234) was used for mouse samples. Relative expression levels for both miRNA and mRNA were calculated using the delta-delta CT method (2^–ΔΔCT) [33].

### Statistical analysis

Data obtained from human tissues are presented as median with interquartile range. Data obtained from cultured cells and from murine experiments are presented as mean±SEM. Normality of each dataset was examined with the Shapiro–Wilk test and homoscedasticity with Levene’s test. When both assumptions were met, inter-group comparisons were performed with a two-tailed Student’s t-test. When either assumption was violated, the non-parametric Mann-Whitney U test was used instead. P-values under 0.05 were considered significant. Correlation analysis was performed using Spearman’s correlation coefficient. All these analyses were performed using the GraphPad Software v8.0 (GraphPad Software, San Diego, USA).

## Results

### Ileal miRNA signature in patients with complicated CD compared with non-IBD subjects

**Fig 1:**
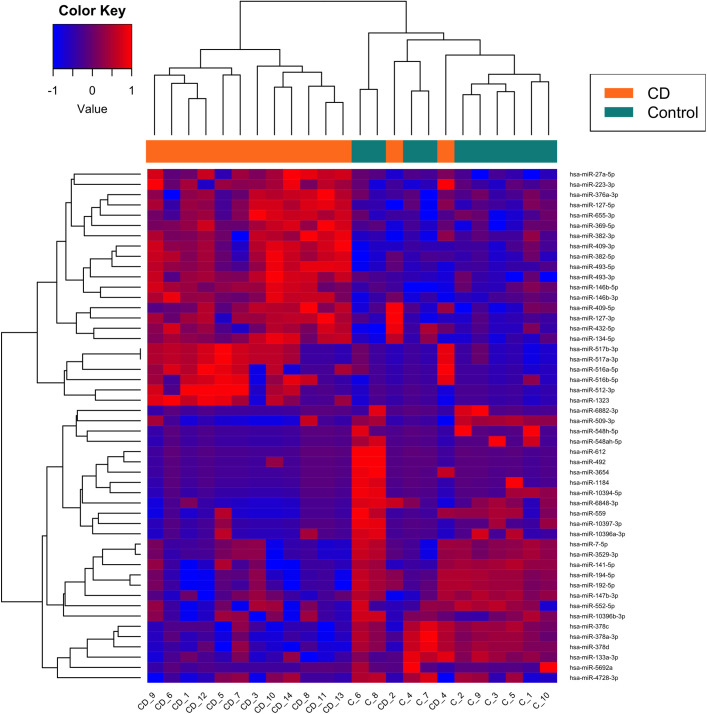
A heatmap showing the top 50 differentially expressed miRNA between the ilea of complicated Patients with CD and non-IBD-subjects as control.

The miRNA sequencing study performed in the ileum from both patients with complicated CD (n = 14) and non-IBD subjects (control ileum, n = 10) shows that 79 miRNAs were significantly down-regulated (FDR < 0.05) and 55 miRNA significantly (FDR < 0.05) up-regulated in the former compared with the later (S1 File). Fig 1 shows the heatmap with the first 50 miRNAs significantly altered.

**Fig 2:**
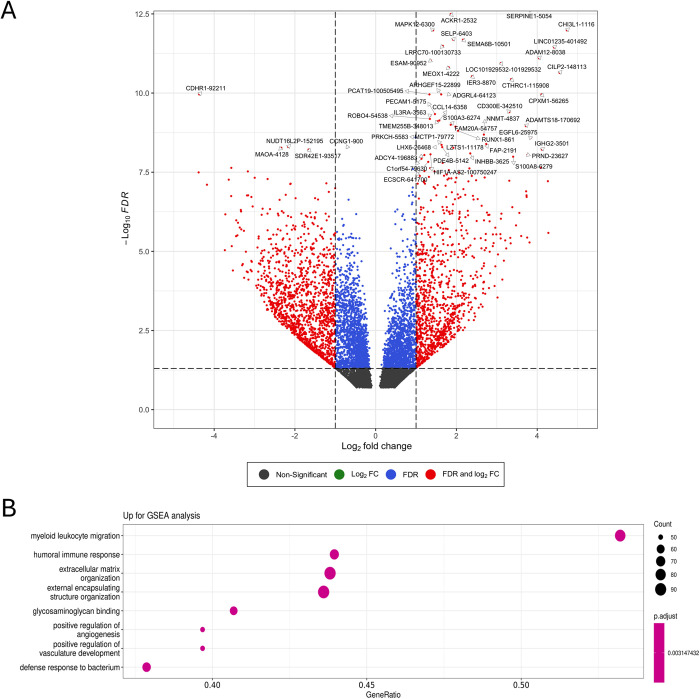
The expression profiles of mRNAs in the ilea of both complicated patients with CD and non-IBD-subjects, as control. **A)** Volcano plot shows the top 50 DEGs between CD (n = 14) and control ileum (n = 10). **B)** Score dot plot showing the enrichment analyses for the differentially up-regulated genes using Gene Ontology (GO) terms database GSEA.

In parallel, the mRNA sequencing study performed in the same samples reveals 8844 differentially expressed genes (DEGs) between the ileum from patients with complicated CD (n = 14) and non-IBD patients (control ileum, n = 10) and 5522 reached statistical significance (FDR < 0.05). Among them, 2487 genes were significantly up-regulated, while 3035 were significantly down-regulated in the fibrotic ileum (S2 File). The volcano plot shows the 50 top DEGs (Fig 2A) and the GO enrichment analysis revealed that genes that were over-expressed in CD lesions were linked to a total of 1137 significantly up-regulated GO-terms (S3 File) from which myeloid leukocyte migration and extracellular matrix organization stands out (Fig 2B). Finally, we correlated genes included in those terms and those miRNAs significantly down-regulated in CD and results (Spearman’s correlation coefficient) point to hsa-miR-378a-3p as one of the miRNAs most significantly and negatively correlated with inflammatory and fibrotic genes (Supplementary S1; Fig A and B)

### The expression of hsa-miR-378a-3p is diminished in the ileum from patients with complicated CD and it is associated with inflammation and fibrosis

**Fig 3:**
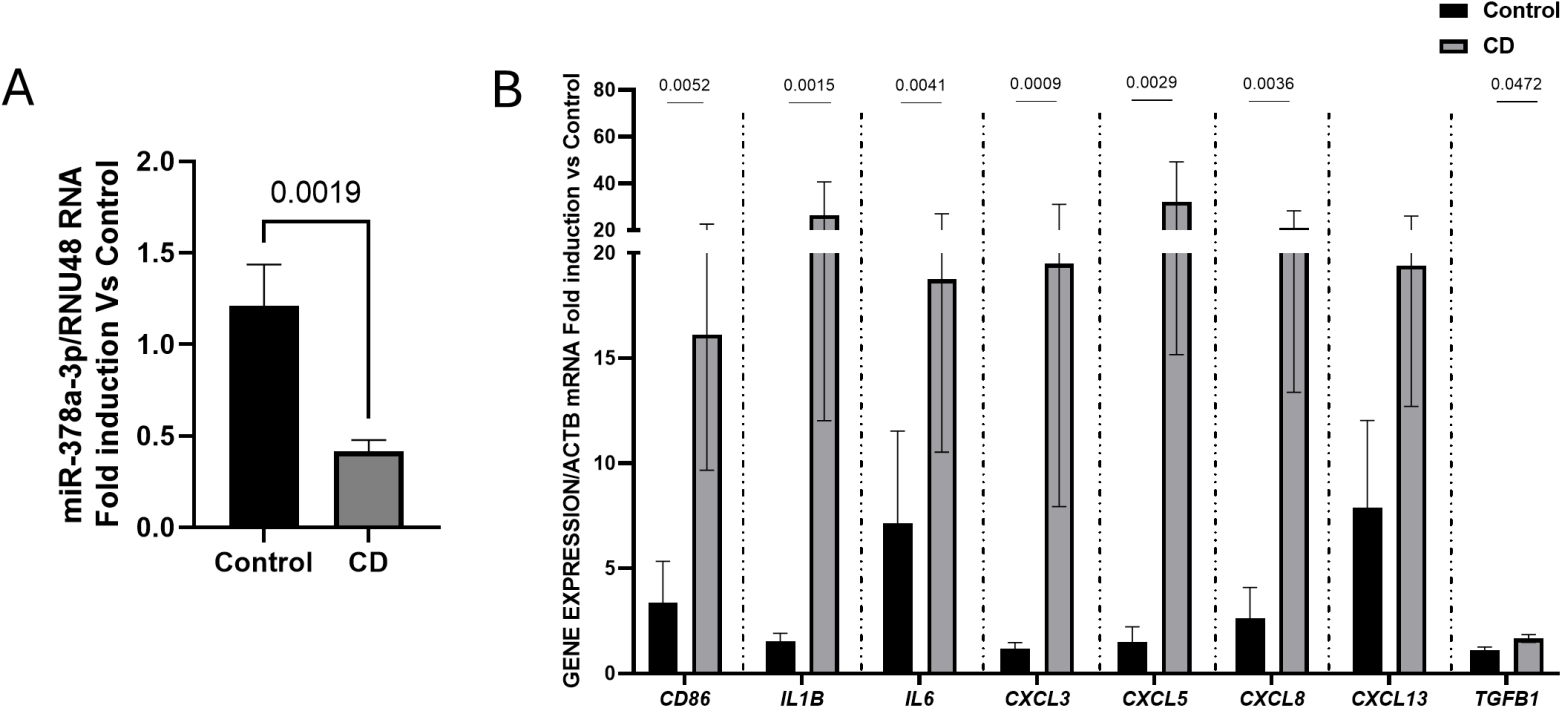
Diminished expression of hsa-miR-378a-3p and increased mRNA expression of inflammatory and fibrotic markers are detected in ileum from patients with complicated CD. **A)** Graph shows the relative expression levels of hsa-miR-378a-3p (RNU48 gene was used to normalize the expression data); **B)** Graph shows the relative mRNA expression levels of selected genes (ACTB gene was used to normalize the expression data). Data show median and the interquartile range of gene expression in control ilea (C, n = 10) and CD ilea (CD, n = 14) and results are expressed as fold induction vs the mean of control samples. Normality of each dataset was examined with the Shapiro–Wilk test and homoscedasticity with Levene’s test. When both assumptions were met, inter-group comparisons were performed with a two-tailed Student’s t-test. When either assumption was violated, the non-parametric Mann-Whitney U test was used instead. The P values under 0.05 are shown.

Next, we validated by RT-PCR the mRNA expression of hsa-miR378a-3p and several inflammatory and fibrotic markers in the human ileum. Results show a significant diminution in the expression of hsa-miR-378a-3p (Fig 3A) and a significant increase in the mRNA expression of pro-inflammatory markers and cytokines (*CD86, IL1B, IL6*) chemokines (*CXCL3, CXCL5, CXCL8, CXCL13*) and pro-fibrotic genes (*TGFB1*), in the ileum from patients with CD compared with control samples (Fig 3B).

### The expression of mmu-miR-378a-3p is diminished in the inflamed and fibrotic intestine of chronic DSS-treated mice: effects of mmu-miR-378a-3p mimics

**Fig 4:**
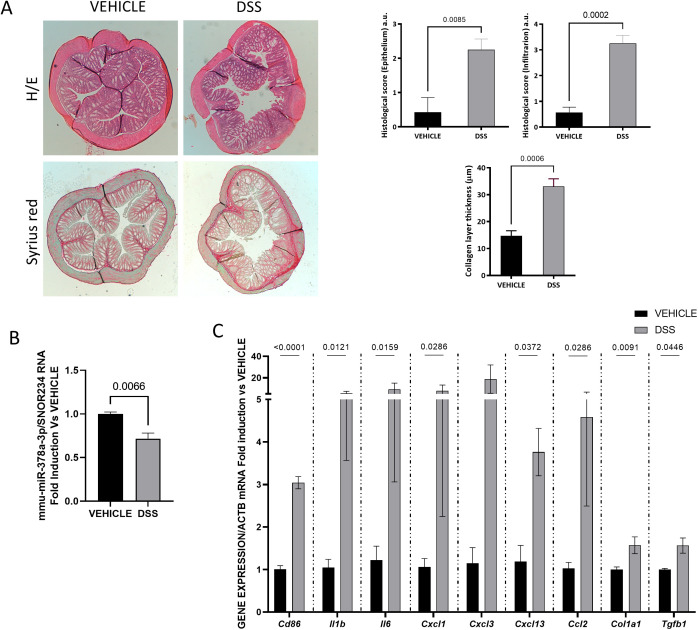
Diminished expression of mmu-miR-378a-3p and increased mRNA expression of inflammatory and fibrotic markers are detected in murine intestine of DSS-treated mice. **A)** Representative 5 µm paraffin-embedded sections of the intestine of vehicle- (n = 7) and DSS-treated mice (n = 8) stained with haematoxylin/eosin and syrius red and graphs show the histological score and the collagen layer thickness in both experimental groups. **B)** Graph shows the relative expression levels of mmu-miR-378a-3p in the colon of vehicle and DSS treated mice (snoRNA234 gene was used to normalize the miRNA expression data). **C)** Graphs show the relative expression levels of several pro-inflammatory and pro-fibrotic genes, analysed by RT-PCR (Actb gene was used to normalize the expression data). In B and C, data show mean and the SEM of gene expression and it is expressed as fold induction vs vehicle-treated mice. Normality of each dataset was examined with the Shapiro–Wilk test and homoscedasticity with Levene’s test. When both assumptions were met, inter-group comparisons were performed with a two-tailed Student’s t-test. When either assumption was violated, the non-parametric Mann-Whitney U test was used instead. The P values under 0.05 are shown.

Colitis was induced in mice by drinking water with 2% DSS, for 7 days, followed by ten days drinking water and this cycle was repeated two times before sacrifice. This murine model is characterized by intestinal architectural distortion and cellular infiltration as well as fibrosis (Fig 4A). In addition, a significant increase in the mRNA expression of inflammatory markers (*cd86),* pro-inflammatory cytokines and chemokines (*Il1b, Il6, Cxcl13, Cxcl1, Ccl2*) and fibrotic markers (*Col1a1, Tgfb1*) was also detected in DSS-treated mice compared with control mice (Fig 4B). The analysis of the RNA expression showed that levels of mmu-miR-378a-3p were lower in the intestine from DSS-treated mice than those detected in control mice (Fig 4C).

**Fig 5:**
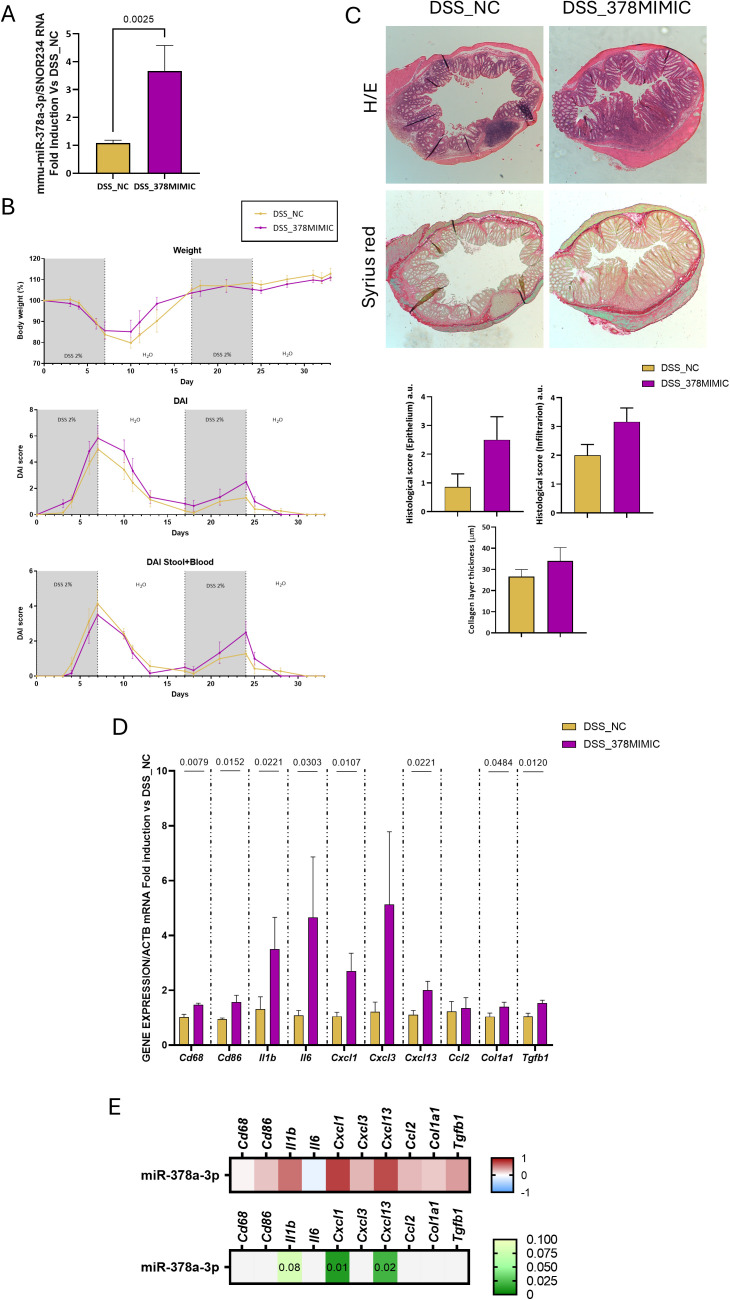
Effects of mmu-miR-378a-3p mimics or NC mimics administration on murine intestinal inflammation and fibrosis induced by DSS. **A)** Graph shows the relative expression levels of mmu-miR-378a-3p in the colon of both NC-DSS-treated mice (n = 7) and mmu-miR-378a-3p mimic-DSS treated mice (n = 6) (snoRNA234 gene was used to normalize the miRNA expression data). **B)** Graphs show the evolution of body weight, the DAI score and the colon length of DSS-treated mice. **C)** Representative 5 µm paraffin-embedded sections of the intestine of both groups after eosin/haematoxylin staining or syrius staining and graphs show the histological score and the collagen layer thickness in both experimental groups. **D)** Graphs show the relative expression levels of several pro-inflammatory and pro-fibrotic genes (Actb gene was used to normalize the expression data). In A,C and D, data show mean and the SEM of gene expression and they are is expressed as fold induction vs NC-treated mice. Normality of each dataset was examined with the Shapiro–Wilk test and homoscedasticity with Levene’s test. When both assumptions were met, inter-group comparisons were performed with a two-tailed Student’s t-test. When either assumption was violated, the non-parametric Mann-Whitney U test was used instead. The P values under 0.05 are shown. **E)** The heatmap on the top show the Spearman’s correlation coefficient (Spearman’s R) between levels of miR-378a-3p and several inflammatory and fibrotic genes in the colon of mice receiving NC mimics or mmu- miR378a-3p mimics. The heatmap on the bottom shows the P value of the correlation.

Next, we analysed the effects of restoring the diminished mmu-miR-378a-3p levels detected in chronic colitis by the intravenous administration of mmu-miR-378a-3p mimics to mice, throughout the experimental period. First, we detected that levels of the expression of mmu-miR-378a-3p were significantly higher in the colon of mice receiving mmu-miR-378a-3p mimics (Fig 5A) than in that of mice receiving NC mimics, 34 days after the beginning of the experiment. As shown in Fig 5B, the administration of mmu-miR-378a-3p mimics did not prevent the significant loss of body weight and the increase in the DAI score when compared with mice that had received NC mimics. In addition, a similar colon length was detected in DSS-treated mice receiving NC mimics or mmu-miR-378a-3p mimics (Fig 5C).

The histological analysis performed in the colon of both NC mimics- and mmu-miR-378a-3p mimics-DSS-treated mice showed an inflammatory infiltrate characterized by presence of lymphocytes, plasmatic cells, macrophages and eosinophils. In addition, architectural distortion and reactive glandular changes with neutrophil infiltration were more often detected in mmu-miR-378a-3p mimics-treated mice than in those receiving NC-mimics. In fact, graphs in Fig 5C show that both the epithelial and the infiltration score were higher in mmu-miR-378a-3p mimics- than in NC mimics- treated mice despite non-significant differences were detected. Furthermore, the RT-PCR analysis reveals that the mRNA expression of inflammatory markers (cd86), pro-inflammatory cytokines and chemokines (Il1b, Il6, Cxcl13, Cxcl1, Ccl2) and fibrotic markers (Col1a1, Tgfb1) in the intestine of DSS-treated mice receiving mmu-miR-378a-3p mimics was significantly higher than that detected in the intestine of DSS-treated mice receiving NC mimics (Fig 5D).

The Syrius staining shows in NC mimics DSS-treated mice an important collagen deposition in both the submucosa and infiltrated in the mucosa and it was also detected in mmu-miR-378a-3p mimics-treated mice (Fig 5C). The quantification of the collagen layer thickness shows similar results (Fig 5C), while the RT-PCR analysis shows a significant increase in the mRNA expression of fibrotic markers (*Col1a1, Tgfb1*) in mmu-miR-378a-3p mimics-treated mice compared with the NC mimics-treated group (Fig 5D). As shown in Fig 5E, results reveal a positive and significant correlation between levels of mmu-miR-378a-3p and inflammatory chemokines and cytokines, which reached statistical significance in the case of cxcl1 and cxcl3.

### The expression of hsa-miR-378a-3p is diminished in M1 macrophages: effects of mimics hsa-miR-378a-3p

**Fig 6:**
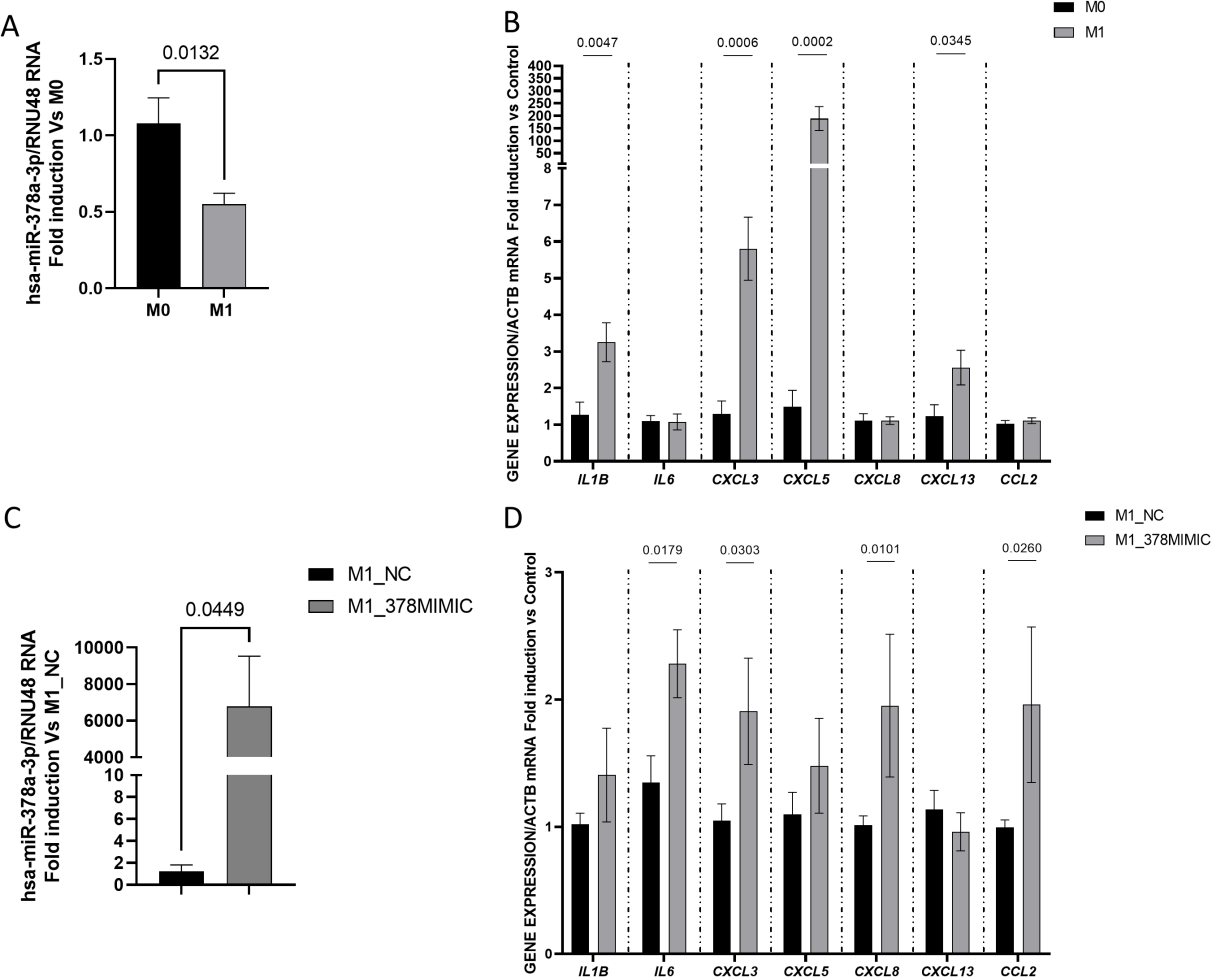
Diminished expression of hsa-miR-378a-3p in pro-inflammatory macrophages and effects of the administration of hsa-miR-378a-3p mimics. **A)** Graph shows the relative expression levels of hsa-miR-378a-3p in non-polarized (M0, n = 8) and M1 polarized macrophages (M1, n = 7) (RNU48 gene was used to normalize the miRNA expression data). **B)** Graph shows the relative expression levels of inflammatory genes in M1 macrophages compared with non-polarized macrophages (ACTB gene was used to normalize the gene expression data). In A and B, data show mean and the SEM and it is expressed as fold induction vs non-polarized macrophages (M0). **C)** Graph shows the relative expression levels of hsa-miR-378a-3p (RNU48 gene was used to normalize the miRNA expression data) in M1 macrophages treated with hsa-miR-378a-3p mimics (n = 7) or NC mimics (n = 7). **D)** Graphs show the relative expression levels of inflammatory genes in M1 macrophages treated with hsa-miR-378a-3p mimics or NC mimics (ACTB gene was used to normalize the gene expression data). In C and D, data show mean and the SEM and it is expressed as fold induction vs the mean of NC mimics-M1 macrophages. Normality of each dataset was examined with the Shapiro–Wilk test and homoscedasticity with Levene’s test. When both assumptions were met, inter-group comparisons were performed with a two-tailed Student’s t-test. When either assumption was violated, the non-parametric Mann-Whitney U test was used instead. The P values under 0.05 are shown.

U937 cells were polarized to M1 macrophages by treatment with LPS + IFN-γ and results reveal a significant decrease in the expression of hsa-miR-378a-3p (Fig 6A) in parallel with a significant increase in the mRNA expression of *IL1B, CXCL3, CXCL5 and CXCL13* (Fig 6B) when compared with non-polarized macrophages. Transfection of M1 macrophages with hsa-miR-378a-3p mimics significantly increased the levels of hsa-miR-378a-3p compared with those transfected with the NC mimics (Fig 6C) and it was associated with a significant increase in the mRNA expression of *IL6, CXCL3, CXCL8* and *CCL2* (Fig 6D). According with the microT-cds database [34], the gene *TRAF3* presents specific miR-378a-p recognition elements, but the analysis of its mRNA expression in M1 macrophages showed non-significant differences between levels detected in NC mimics and hsa-mir-378a-3p mimics treated cells (Supplementary S2Fig).

### The expression of hsa-miR-378a-3p is not significantly modified in activated fibroblasts: effects of hsa-miR-378a-3p mimics

**Fig 7:**
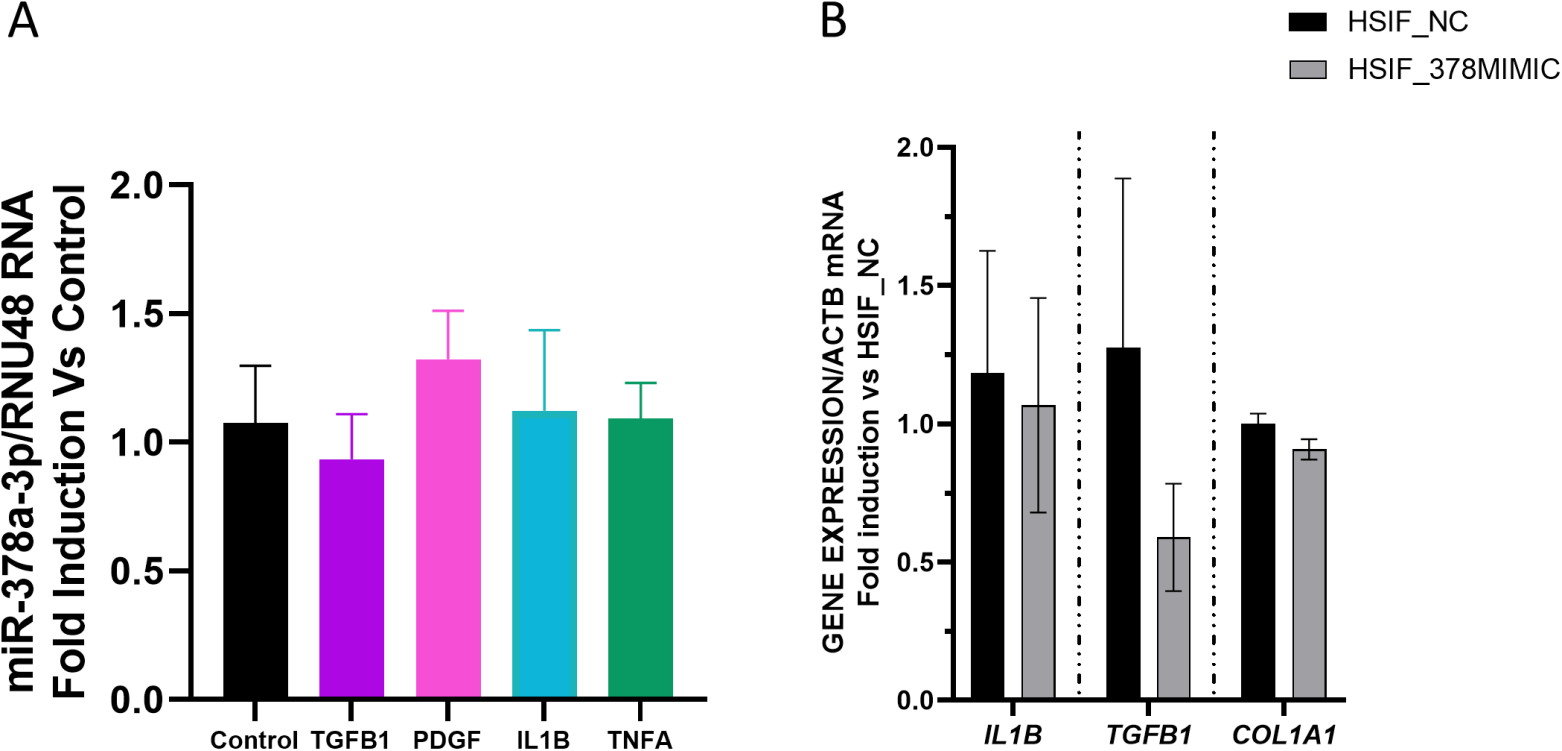
Levels of hsa-miR-378a-3p in activated fibroblasts and effects of the administration of hsa-miR-378a-3p mimics. **A)** Graph shows the relative expression levels of hsa-miR-378a-3p in fibroblasts treated with vehicle (n = 4), DLL4 (n = 4), TNFα (n = 3), IL1β (n = 4), TGFβ1 (n = 4) and PDGF (n = 3); (RNU48 gene was used to normalize the miRNA expression data). Data show mean and the SEM of gene expression and it is expressed as fold induction vs control. **B)** Graph shows the relative expression levels of selected genes (*IL1B*, *TGFB1* and *COL1A1*) in fibroblasts treated with hsa-miR-378a-3p mimics (n = 3) or NC mimics (n = 3) (ACTB gene was used to normalize the expression data). Data show mean and the SEM of gene expression and it is expressed as fold induction vs the mean of NC mimics-treated fibroblasts. Normality of each dataset was examined with the Shapiro–Wilk test and homoscedasticity with Levene’s test. When both assumptions were met, inter-group comparisons were performed with a two-tailed Student’s t-test. When either assumption was violated, the non-parametric Mann-Whitney U test was used instead.

Treatment of fibroblasts with different ligands, IL1β, TGFβ, PDGFB, or TNFα, at doses previously shown to activate these cells [20] failed to significantly modify the expression of hsa-miR-378a-3p in human small intestinal fibroblasts (Fig 7A).

Transfection of fibroblasts with hsa-miR-378a-3p mimics failed to significantly modify the mRNA expression of *IL1B, TGFB1* or *COL1A1,* when compared with NC mimics-treated fibroblasts (Fig 7B).

## Discussion

The present study shows the downregulation of miR-378a-3p expression in human ileum of patients with complicated CD, M1 polarized macrophages and intestine from DSS-treated mice and demonstrates that restoring the intestinal miR-378a-3p levels did not prevent inflammation and fibrosis in murine intestine while exacerbated the expression of inflammatory and fibrotic markers.

Our sequencing study identifies those mRNA and miRNA differentially expressed between the fibrotic ileum of patients with complicated CD and non-IBD subjects. The enrichment analysis of the mRNA seq data shows that genes involved in extracellular matrix organization and leukocyte migration are among the top up-regulated. To better understand the potential epigenetic regulation of these genes, we correlated them with those miRNAs that were down-regulated in the ileum of patients with CD and results points to hsa-miR-378a-3p. Previous studies have reported a reduced expression of this miRNA in the inflamed mucosa of patients with ulcerative colitis [35] and liver fibrosis [36]; however, little is known about the expression of this miRNA in ileal complicated CD and only one study described diminished hsa-miR-378a-3p levels in feces of non-complicated CD patients [37]. The validation by RT-PCR of both mRNA and miRNA suggest that hsa-miR-378a-3p acts as an epigenetic modulator of inflammatory and/or fibrotic genes.

To analyse the relevance of miR-378a-3p, we set out a murine model of chronic colitis by DSS administration to mice. Of interest, our results also revealed a significant reduction in the expression of mmu-miR-378a-3p in the inflamed and fibrotic murine colon. This diminution runs in parallel with increased mRNA expression of inflammatory and fibrotic markers thus reinforcing that intestinal inflammation and fibrosis are associated to decreased miR-378a-3p expression. The restoration of the diminished levels of this miRNA by the intravenous administration of mmu-miR-378a-3p mimics to mice failed to prevent inflammation and fibrosis. In contrast, we found a significant increase in the expression of inflammatory cytokines and chemokines [38] in the intestine of mmu-miR-378a-3p mimics- compared with that of NC mimics-treated mice. In this line, the histological analysis revealed a higher infiltration of leukocytes and its characterization by RT-PCR demonstrates a significant increased expression of M1 macrophage markers in mmu-miR-378a-3p mimics treated mice. Based on previous studies showing the up-regulation of miR-378a-3p expression in macrophages polarized towards a regulatory phenotype by IL4 [39,40], we used isolated macrophages and polarized them towards an inflammatory phenotype with LPs and IFNγ [29,41]; results show a significant reduction in miR-378a-3p expression in parallel with increased expression of cytokines and chemokines in M1 macrophages compared with non-polarized cells. These observations are in accordance with previous studies showing both the down-regulation of miR-378a-3p by TNFα in colonocytes [35] and changes in the expression of inflammatory markers inversely associated with miR-378a-3p levels [42]. In addition to that observed in murine intestine, our data also show that the administration of miR-378a-3p mimics to M1 macrophages, increased the expression of several cytokines and chemokines which point to macrophages as one of the cell types involved in the up-regulation of cytokines induced by miR-378a-3p mimics in murine intestine. In contrast, our in vitro data do not support a direct effect of mir-378-3p mimics on fibroblasts, since we did not detect changes in the expression of cytokines and fibrotic markers by the transfection of miR-378a-3p mimics and different stimuli failed to modulate miR-378a-3p expression in these cells.

An important limitation of the study has been the failure in identifying the direct target/s of hsa-miR-378a-3p that might explain the effects of mmu-miR-378a-3p mimics on inflammation. The increase in the expression of several cytokines induced by miR-378a-3p mimics led us to think in a negative regulator of the transcription of these genes, as a potential direct target of hsa-miR-378a-3p. A search in the MicroT-cds database [34], showed that the TNF receptor, TRAF3, which has been shown to down-regulate transcription factors involved in inflammatory pathways such as NFkB and CREB1 [43–46], presents specific miR-378a-p recognition elements (MREs) located in 3’-UTR region. Unfortunately, our results failed to demonstrate changes in *TRAF3* mRNA expression by transfection of macrophages with hsa-miR-378a-3p mimics. Further studies are required to identify the gene/s target of miR-378a-3p that may explain the increased cytokines expression detected.

In conclusion, our study reveals diminished expression of miR-378a-3p in inflammatory and fibrotic intestine of both patients with complicated CD and murine chronic colitis; The administration of miR-378a-3p mimics restores the intestinal miR-378a-3p levels in DSS-treated mice but it failed to prevent inflammation and fibrosis while increased the expression of inflammatory markers in both *in vitro* and *in vivo* models.

## Supporting information

S1 Table Patients´s characteristics. (DOCX)

S2 Table Primer sequences of specific PCR products for each gene analyzed. (DOCX)

S1 Fig A correlation analysis between transcripts obtained from both the mRNA and miRNA sequencing analysis in ileal resections from control and patients with CD. The heatmaps on the top show the Spearman’s correlation coefficient (Spearman’s R) of those miRNAs downregulated in CD and genes included in the term myeloid leukocyte migration (A) and extracellular matrix organization (B). The heatmaps on the bottom shows the P value of each correlation analysis.
(TIFF)

S2 Fig Graph shows the relative mRNA expression levels of TRAF3 in M1 macrophages treated with hsa-miR-378a-3p mimics or NC mimics (ACTB gene was used to normalize the gene expression data). Data show mean and the SEM and it is expressed as fold induction vs the mean of NC mimics-M1 macrophages.
(TIF)

S1 File Excel showing the differentially expressed miRNA between affected ileum from patients with CD and control ileum obtained from non-IBD patients. (XLSX)

S2 File Excel showing the differentially expressed mRNA between affected ileum from patients with CD and control ileum obtained from non-IBD patients. (XLSX)

S3 File Excel showing the GO enrichment analysis of the mRNA sequencing study. (XLSX)
