# Supplementary material for: Down-regulation of MIR-378A-3P expression associated with inflammation: The effects of restoring its levels
Source: PLoS One. 2025 Aug 11;20(8):e0329685. doi: 10.1371/journal.pone.0329685 (PMC12338774; doi:10.1371/journal.pone.0329685)
Supplement: S1 Table — (DOCX) [file pone.0329685.s001.docx]

**Supplementary Table 1.** Patients’ characteristics and dates of surgical resections accessed for research

| **Sequencing Samples** | ***CD*** | ***C*** | | |
| --- | --- | --- | --- | --- |
| ***Number of patients*** | 14 | 10 | | |
| ***Age*** | 49,64 (20-77) | 68,9 (41-88) | | |
| ***Gender*** | 50% M/50% F | 40% M/60% F | | |
| ***Localization*** | | | | |
| Terminal ileum | 14 | 10 | | |
| ***Behaviour*** | |  | |  |
| *B2* | *6* |  |  |  |
| *B3* | *8* |  |  |  |
| ***Concomitant medication*** | |  |  |  |
| *CORTICOID* | 1 |  |  |  |
| *AZATHIOPRINE* | 1 |  |  |  |
| *BIOLOGICAL THERAPY* |  |  |  |  |
| *Ustekinumab* | 4 |  |  |  |
| *Anti-TNFα* | 4 |  |  |  |
| *Vedolizumab* | 1 |  |  |  |
| *NO TREATMENT* | 2 |  |  |  |
| *UNKNOWN* | 1 |  |  |  |

| **CD1** | 13/05/21 | **C1** | 25/05/2021 |
| --- | --- | --- | --- |
| **CD2** | 20/05/21 | **C2** | 03/06/2021 |
| **CD3** | 25/05/21 | **C3** | 18/06/2021 |
| **CD4** | 27/05/21 | **C4** | 13/09/2021 |
| **CD5** | 11/06/21 | **C5** | 16/09/2021 |
| **CD6** | 06/09/21 | **C6** | 14/01/2022 |
| **CD7** | 07/09/21 | **C7** | 02/05/2022 |
| **CD8** | 10/09/21 | **C8** | 30/05/2022 |
| **CD9** | 04/11/21 | **C9** | 20/10/2022 |
| **CD10** | 21/02/22 | **C10** | 26/10/2022 |
| **CD11** | 25/02/22 |  |  |
| **CD12** | 03/03/21 |  |  |
| **CD13** | 04/04/22 |  |  |
| **CD14** | 09/12/22 |  |  |
