# Supplementary material for: Down-regulation of MIR-378A-3P expression associated with inflammation: The effects of restoring its levels
Source: PLoS One. 2025 Aug 11;20(8):e0329685. doi: 10.1371/journal.pone.0329685 (PMC12338774; doi:10.1371/journal.pone.0329685)
Supplement: S2 Table — (DOCX) [file pone.0329685.s002.docx]

**Supplementary Table 2.** Primer sequences of specific PCR products for each gene analyzed

| Gene ID | Gene Name | Sense (5’-3’) | Antisense (5’-3’) |
| --- | --- | --- | --- |
| 3576 | CXCL8 | AGAGACAGCAGAGCACACAAG | AATTTGGGGTGGAAAGGTTTGG |
| 10563 | CXCL13 | GCTTGAGGTGTAGATGTGTCC | GAGCAGGGATAAGGGAAGAATG |
| 4170 | MCL1 | GAGGAGGACGAGTTGTACCG | TCCTGCCCCAGTTTGTTACG |
| 1281 | COL3A1 | CGCCCTCCTAATGGTCAAGG | TTCTGAGGACCAGTAGGGCA |
| 596 | BCL2 | TCTTTGAGTTCGGTGGGGTC | GCCGTACAGTTCCACAAAGG |
| 942 | CD86 | GTCTGTCCACCCCATCAAC | GTATCACCAAAACCCCTCCC |
| 3553 | IL1B | TTTTTGCTGTGAGTCCCGGAG | TTTTTGCTGTGAGTCCCGGAG |
| 3569 | IL6 | AGTGAGGAAGCCAGAGC | ATTGTGGTTGGGTCAGGGG |
| 2921 | CXCL3 | CGCCCAAACCGAAGTCATAG | TCATTTTCAGCTCTGGTAAGGG |
| 6374 | CXCL5 | CAGTAATCTGCAAGTGTTCGCC | GTTTTCCTTGTTTCCACCGTCC |
| 4360 | CD206 | CTTTGGACGGATGGACGAGG | CAAGGAAGGGTCGGATCGTG |
| 383 | ARG1 | AGGGACAGCCACGAGGAGGG | AGTTTCTCAAGCAGACCAGCCTTTC |
| 7040 | TGFB1 | AGCAACAATTCCTGGCGATAC | CGGTAGTGAACCCGTTGATG |
| 6347 | CCL2 | CATAGCAGCCACCTTCATTCC | CACAGCTTCTTGGGACACTGG |
| 1116 | CHI3L1 | GGAATGATGTGACGCTCTACGG | CTGGGTGTTGGAGGCTATCTTG |
| 60 | ACTB | GGACTTCGAGCAAGAGATGG | AGCACTGTGTTGGCGTACAG |
| 12514 | Cd68 | GTTCACCTTGACCTGCTCTC | TTGATTGTCGTCTGCGGG |
| 12524 | Cd86 | GCACGGACTTGAACAACCAG | CCTTTGTAAATGGGCACGGC |
| 16176 | Il1b | TGCCACCTTTTGACAGTGATG | ATGTGCTGCTGCGAGATTTG |
| 16193 | Il6 | GAGTCCTTCAGAGAGATACAGAAAC | TGGTCTTGGTCCTTAGCCAC |
| 14825 | Cxcl1 | CGAAGTCATAGCCACACTCAAG | TCTCCGTTACTTGGGGACAC |
| 330122 | Cxcl3 | ACCCAGACAGAAGTCATAGCC | GCAGGTAAAGACACATCCAGAC |
| 55985 | Cxcl13 | TCGGATTCAAGTTACGCCCC | ATTTGGCACGAGGATTCACAC |
| 20296 | Ccl2 | ACCTGCTGCTACTCATTCACC | CCCATTCCTTCTTGGGGTCAG |
| 17533 | Cd206 | TGTGGAGCAGATGGAAGGTC | TGTCGTAGTCAGTGGTGGTTC |
| 12655 | Ym1 | AGAAGCAATCCTGAAGACACC | GCATTCCAGCAAAGGCATAG |
| 11846 | Arg1 | GTGGGGAAAGCCAATGAAGAG | TCAGGAGAAAGGACACAGGTTG |
| 16818 | Lck | TGTGGTCCTTCGGGATCTTG | TTGTCAGGTCTCACCATGCG |
| 12842 | Col1a1 | CAGGCTGGTGTGATGGGATT | AAACCTCTCTCGCCTCTTGC |
| 21803 | Tgfb1 | GCGGACTACTATGCTAAAGAGG | TCAAAAGACAGCCACTCAGG |
| 11489 | Adam12 | AGTGTCTTCGGCGTTCACAAG | CACCACAAATCCAGCAGCAAG |
| 17390 | Mmp2 | GTGCGACCACAACCAACTAC | GTCAGTATCAGCATCGGGGG |
| 11461 | Actb | GCCAACCGTGAAAAGATGACC | GAGGCATACAGGGACAGCAC |
