# Supplementary figures and images for: Down-regulation of MIR-378A-3P expression associated with inflammation: The effects of restoring its levels
Source: PLoS One. 2025 Aug 11;20(8):e0329685. doi: 10.1371/journal.pone.0329685 (PMC12338774; doi:10.1371/journal.pone.0329685)

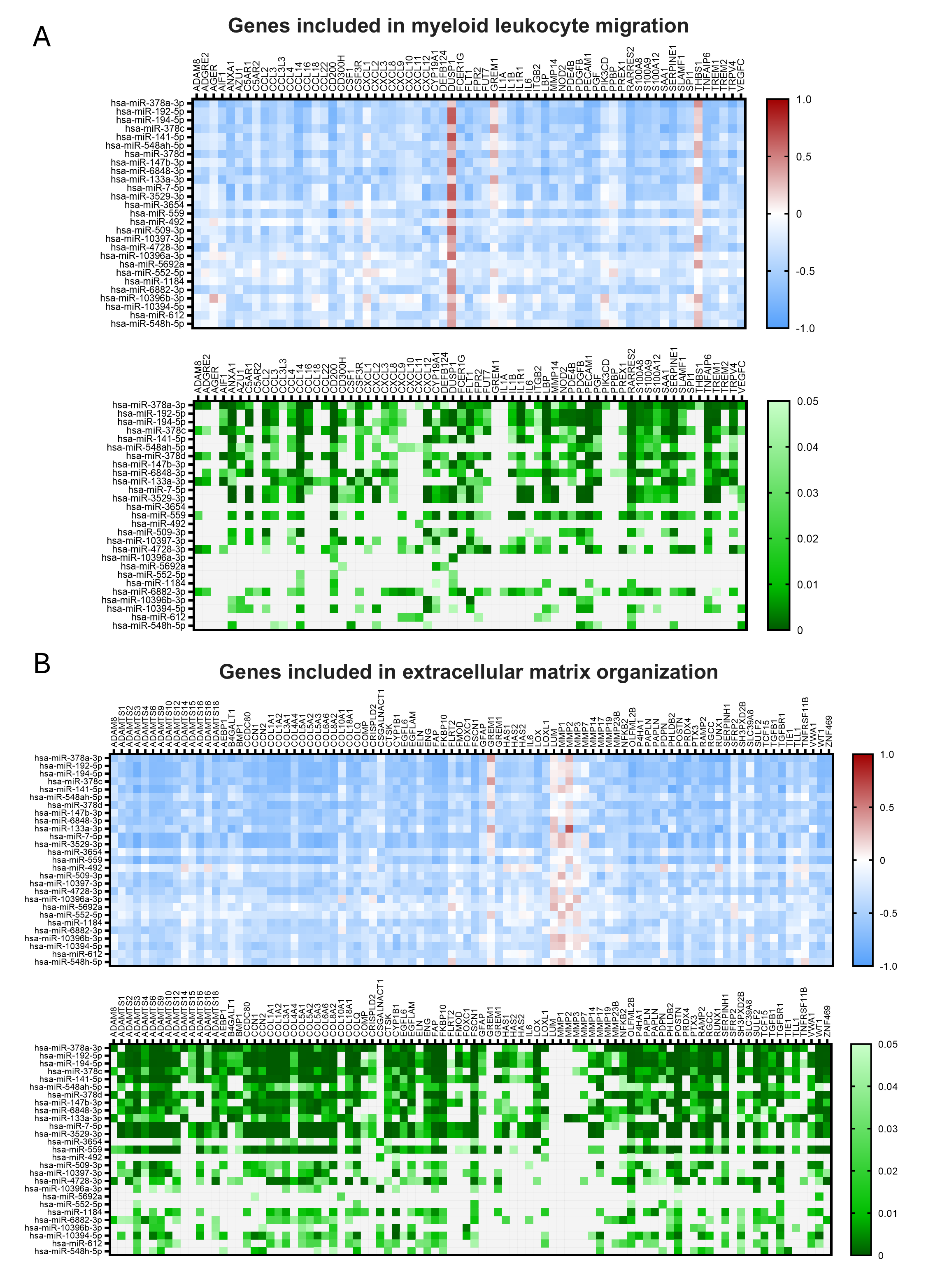

Supplement: S1 Fig — The heatmaps on the top show the Spearman’s correlation coefficient (Spearman’s R) of those miRNAs downregulated in CD and genes included in the term myeloid leukocyte migration (A) and extracellular matrix organization (B). The heatmaps on the bottom shows the P value of each correlation analysis. (TIFF) [file pone.0329685.s003.tiff]

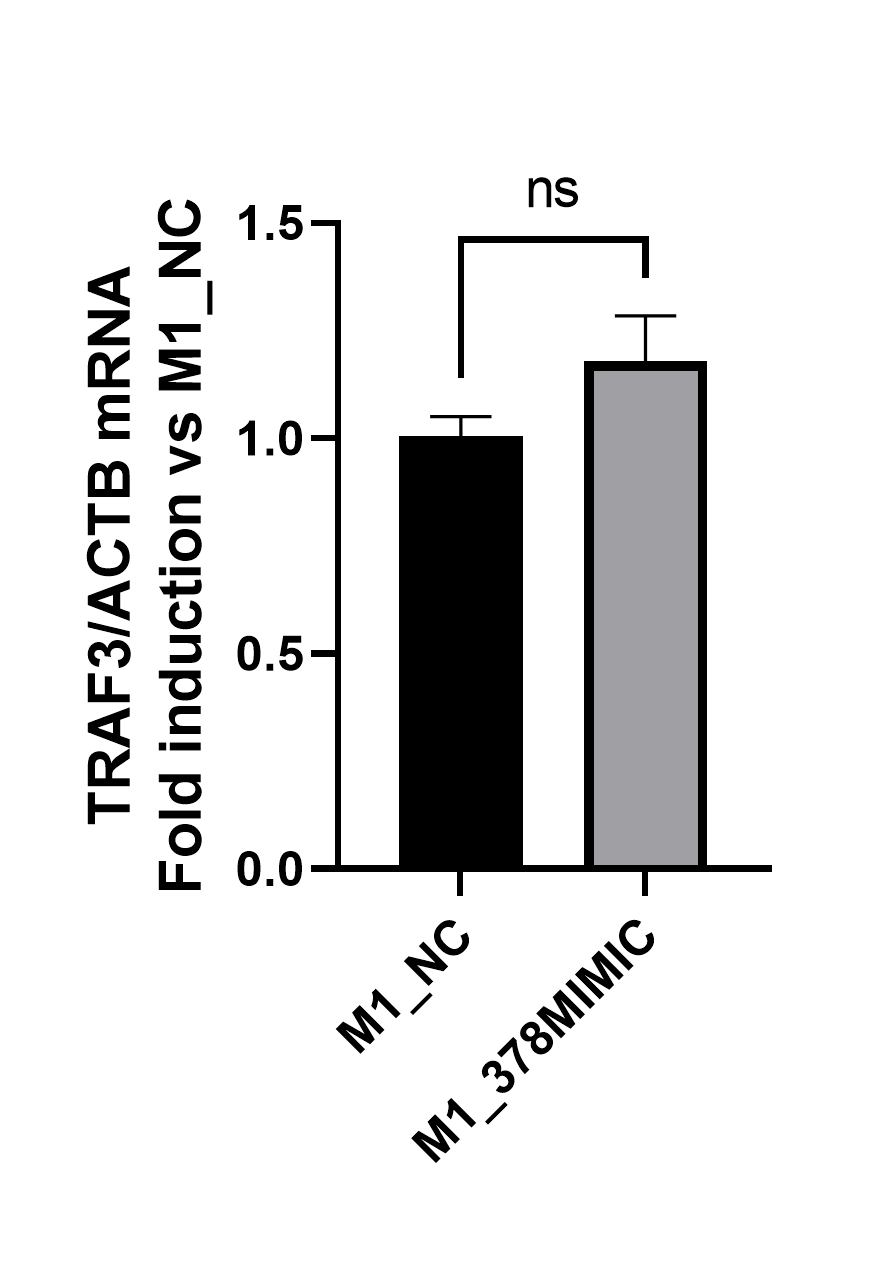

Supplement: S2 Fig — Data show mean and the SEM and it is expressed as fold induction vs the mean of NC mimics-M1 macrophages. (TIF) [file pone.0329685.s004.tif]
